# Assessment of microbiological correlates and immunostimulatory potential of electron beam inactivated metabolically active yet non culturable (MAyNC) *Salmonella* Typhimurium

**DOI:** 10.1371/journal.pone.0243417

**Published:** 2021-04-16

**Authors:** Chandni Praveen, Sohini S. Bhatia, Robert C. Alaniz, Robert E. Droleskey, Noah D. Cohen, Palmy R. Jesudhasan, Suresh D. Pillai

**Affiliations:** 1 National Center for Electron Beam Research-an International Atomic Energy Agency (IAEA) Collaborating Centre for Electron Beam Technology, Texas A&M University, College Station, TX, United States of America; 2 Department of Microbial Pathogenesis and Immunology, Texas A&M University Health Science Center, College Station, TX, United States of America; 3 Food and Feed Safety Research Unit, Southern Plains Agricultural Research Center, USDA-ARS, College Station, TX, United States of America; 4 Department of Large Animal Clinical Sciences, College of Veterinary Medicine and Biomedical Sciences, Texas A&M University, College Station, TX, United States of America; 5 Poultry Production and Product Safety, USDA-ARS, University of Arkansas, Fayetteville, AR, United States of America; Universitatsklinikum Erlangen, GERMANY

## Abstract

This study investigates the microbiological and immunological basis underlying the efficacy of electron beam-inactivated immune modulators. The underlying hypothesis is that exposure to eBeam-based ionization reactions inactivate microorganisms without modifying their antigenic properties and thereby creating immune modulators. The immunological correlates of protection induced by such eBeam based *Salmonella* Typhimurium (EBST) immune modulators in dendritic cell (DC) (*in vitro*) and mice (*in vivo*) models were assessed. The EBST stimulated innate pro inflammatory response (TNFα) and maturation (MHC-II, CD40, CD80 and CD86) of DC. Immuno-stimulatory potential of EBST was on par with both a commercial *Salmonella* vaccine, and live *Salmonella* cells. The EBST cells did not multiply under permissive *in vitro* and *in vivo* conditions. However, EBST cells remained metabolically active. EBST immunized mice developed *Salmonella*-specific CD4+ T-cells that produced the Th1 cytokine IFNγ at a level similar to that induced by the live attenuated vaccine (AroA^-^ ST) formulation. The EBST retained stable immunogenic properties for several months at room temperature, 4°C, and -20°C as well as after lyophilization. Therefore, such eBeam-based immune modulators have potential as vaccine candidates since they offer the safety of a “killed” vaccine, while retaining the immunogenicity of an “attenuated” vaccine. The ability to store eBeam based immune modulators at room temperature without loss of potency is also noteworthy.

## Introduction

Vaccines that most closely mimic the pathogenic organism’s form and function, induce superior protective immune responses [1]. Traditionally, live attenuated vaccines are considered more effective compared to inactivated/ killed or subunit vaccines, because of their ability to preserve the integrity of immunological epitopes thereby eliciting enhanced cell mediated immune responses [2, 3]. However, safety concerns limit the utilization of live/attenuated preparations as vaccines, especially considering the increasing number of chronically immunocompromised individuals, with a high risk of contracting vaccine-induced diseases after immunization [4, 5]. Formaldehyde, glutaraldehyde, or heat are commonly used to develop inactivated or “killed” vaccines [6]. However, such vaccine formulations can suffer from lot-to-lot variation due to inadequate mixing of the inactivating chemicals as well as changes in the immunological properties due to damaged antigens [7]. Moreover, there is a growing need to eliminate use of the chemicals in vaccine production [8]. Hence, there is great value for exploring alternate technologies to create formulations that combine the strong immune response of live attenuated vaccines and the safety profile of inactivated vaccines.

Electron beam (eBeam) technology uses linear accelerators to generate a highly planar stream of energetic electrons from commercial electricity [9, 10]. This technology is currently employed worldwide for medical device sterilization, food pasteurization, spice decontamination, phytosanitary treatment of fresh fruits and vegetables, and polymer crosslinking. eBeam irradiation causes multiple double strand breaks in bacterial DNA resulting in bacterial cells inability to multiply [11, 12]. There are earlier reports of using ionizing radiation from radioactive isotopes such as cobalt-60 and cesium-137 for developing vaccines [13–15]. The use of eBeam irradiation for vaccine development is relatively novel and very few studies have been reported. We previously reported the efficacy of eBeam-based vaccines against *S*. Typhimurium [16] and *S*. Enteritidis [17] in imparting protection against *Salmonella* challenge and have patented the technology (*US patent 8*,*173*,*139*). Since then there have been other reports of employing eBeam for developing such immune modulators. The immunological correlates protection of such immune modulators have not been examined in detail. The immune response of *S*. Typhimurium is widely studied using mice models [3, 18, 19]. Studies have revealed that live and heat killed *Salmonella* trigger differential immune effector pathways, and live attenuated *Salmonella* are immunogenically superior to inactivated vaccines due to their ability to induce cell-mediated immune response [2, 20–23]. The objective of this study was to determine whether eBeam inactivated *Salmonella* cells induced comparable immune responses as live Salmonella. The underlying hypothesis was that eBeam irradiation will inactivate bacterial cells without altering their antigenic characteristics and therefore elicit defined immunological responses similar to a live attenuated vaccine. To test this hypothesis, we analyzed the microbiological correlates and assessed immune-stimulatory potential of eBeam-inactivated *S*. Typhimurium in comparison to live (attenuated) (AroA) and heat-killed *S*. Typhimurium (HKST) preparations using *in vitro* (dendritic cells) and in vivo (mouse model) studies.

## Materials and methods

### Bacterial strains and experimental animals

*Salmonella enterica* serovar Typhimurium strain ST14028 was used as the strain for the immune modulator preparation (EBST) preparation. *S*. Typhimurium strain 14028, aroA^-^ (del-STM0978::KanR; aroA null) was used as the live attenuated bacterial (AroA) vaccine [24] control in the mice studies. The cells were grown overnight at 37°C in Tryptic Soy Broth (TSB), washed twice in PBS, and suspended in PBS at a cell density of approximately 10^9^ CFU/ml. To prepare the heat-killed *S*. Typhimurium (HKST) preparation, aliquots of the bacterial suspension were heat inactivated at 70°C for 1 h. Six to eight weeks old female C57BL/6J mice (Jacksons Laboratory, Bar Harbor, Maine) used in this study were housed in specific pathogen free conditions and cared for according to Texas A&M University Institutional Animal Care and Use Committee guidelines (Protocol number: 2015–0010). This protocol was approved by the Texas A&M University Institutional Animal Care and Use Committee. For bone marrow harvesting, mice were euthanized by Carbon di oxide asphyxiation causing rapid anesthesia, and death occurring with in 2–3 minutes. For experiments involving organ harvest, cervical dislocation was performed manually that resulted in euthanasia with in approximately 10–15 seconds.

### eBeam inactivation

All Electron beam (eBeam) irradiation treatments were performed at the National Center for Electron Beam Research at Texas A &M University in College Station, Texas using a 10 MeV, 15 kW, linear accelerator. For eBeam irradiation studies, aliquots of *S*. Typhimurium cells were placed in sealable plastic pouches as previously described [25]. Cells were exposed to defined eBeam doses (0.2 to 8 kGy). The delivered eBeam doses were measured using alanine dosimetry as previously described [25]. These measured doses were used for data plotting and calculation of inactivation kinetics. The inability of lethally inactivated bacteria to multiply was verified using *in vitro* and *in vivo* approaches. The eBeam-inactivated cells were incubated in Tryptic Soy Broth (TSB) at 37°C and room temperature (25°C) for up to 10 days and scored for visual evidence of cell multiplication. The absence of multiplication was verified by plating 200 μl on TSA plates. Direct microscopic counts of EBST were also made using the Petroff-Hausser counting chamber at defined times post irradiation. Based on the above verification studies, an eBeam dose of 7 kGy was chosen for the EBST immune modulator formulation. The lack of culturability of EBST was tested *in vivo* using C57BL/6J mice, an innately susceptible mice model for *S*. Typhimurium [26]. Three groups of mice (5 mice/ group) were orally gavaged with 1 x 10^8^ CFU of EBST (EBST mice), 1 x 10^8^ CFU live *Salmonella* Typhimurium (live ST mice) and 200 μl of PBS (sham mice). The mice were observed for presence of any disease symptoms and mortality. Fecal samples (~0.1g/ mouse) were collected from individual mice on days 3, 7, 10 and 14, post oral gavage. Homogenized fecal samples were plated on Xylose Lysine Deoxycholate (XLD) plates, selective for *Salmonella* sp.. Mice were euthanized 14 days post oral gavage. The liver, spleen, mesenteric lymph nodes (MLN), and cecum from the EBST fed mice were homogenized and plated on XLD plates.

### Cell surface analysis

The physical integrity of EBST cell membranes was verified using the LIVE/DEAD^™^ staining assay. This assay was performed using the LIVE/DEAD^™^ BacLight™ bacterial viability kit for microscopy (Molecular Probes, Eugene, OR). The green fluorescent SYTO^®^9 and the red fluorescent propidium iodide nucleic acid stains were used to stain the EBST, HKST and live ST cell preparations as per manufacturer’s protocol. The stained cells were examined using fluorescence microscopy. Cells with intact cell membranes would fluoresce green and cells with compromised cell membranes would fluoresce reddish orange. To verify whether ultrastructural changes occurred after eBeam irradiation, the EBST, live and HKST cells were examined using Scanning Electron microscopy (SEM) and Transmission electron microscopy (TEM) as previously described [17].

### Immunoreactivity profiles

The *Salmonella* specific immunoreactivity profile of EBST was compared with live ST and HKST using immunoblotting. After eBeam irradiation, bacterial samples were stored at room temperature, 4°C, -20°C and lyophilized conditions. After one month of storage at indicated conditions, total soluble protein was extracted using B-PER Bacterial protein extraction reagent (Thermo Scientific, Rockford, IL). Proteins extracted from an overnight culture of *S*. Typhimurium and HKST were used for comparison. The extracted proteins (20 μg per sample) were separated under reducing conditions on an SDS PAGE gradient (4–20%) gel (Precise Protein Gel, Thermo Scientific) and subsequently transferred to PVDF membrane for western blot analysis [27]. The membrane was blocked (overnight) at 4°C using 5% BSA in Tris Buffered Saline, with 0.05% Tween 20 (TBST) (Sigma Aldrich, St Louis, MO). The membrane was washed twice with TBST and incubated with primary antibody for 2 h at room temperature. Immune serum from mice, previously immunized with *Salmonella* Typhimurium (a kind gift from Dr. R.C. Alaniz, Texas A&M Health Science Center, College Station, TX) was used as primary antibody (1/1000 dilution). Following incubation, the membrane was washed 4 times in TBST and incubated at room temperature for 1 h, with 1/ 20,000 dilution of alkaline phosphatase conjugated sheep anti mouse IgG F(ab`)2 fragment (Sigma Aldrich, St Louis, MO). Further, the membrane was washed 4 times with TBST and developed using chromogenic reagent, NCP/ BCIP (Thermo Scientific, Rockford, IL). Excess chromogenic reagent was washed off using deionized water and the membrane was dried and digitally recorded.

### Metabolic activity

The metabolic activity of EBST was assessed using Alamar Blue® reagent (Life Technologies, Carlsbad, CA). Alamar Blue® system incorporates a cell permeable, non-fluorescent, blue redox indicator, resazurin. Metabolically active cells maintain a reduced cell environment that converts resazurin to resorufin (red fluorescent compound). The increase in fluorescence can be quantitatively measured to determine the metabolic activity of cells. After eBeam irradiation, bacterial samples were stored at 4°C and the persistence of metabolic activity in the eBeam–inactivated *S*. Typhimurium was monitored for 10 days. Live *S*. Typhimurium and HKST were used as controls. Ninety microliters of EBST, HKST and live ST samples were mixed with 10μl of Alamar blue reagent and incubated at 37°C for 1 h. Fluorescence was measured at 530–560 nm excitation wavelength and 590 nm emission wavelength using Wallac 1420 VICTOR 2™ plate reader (PerkinElmer, Waltham, MA).

Biochemical assays were performed to confirm the presence of metabolic activity in eBeam-inactivated *S*. Typhimurium. The ability of bacteria to ferment carbohydrates in media was tested by incubating EBST and HKST in Phenol Red Broth supplemented with sucrose at 37°C for 2 days. Overnight cultures of live ST and *E*. *coli* were used as controls. The color change in the media, turbidity and gas production as a result of bacterial metabolism were monitored. Presence of catalase enzyme in the inactivated cells was detected using the catalase test. On clean glass slides, EBST and live ST cells were smeared and a drop of hydrogen peroxide was added. Bubble formation from the bacterial preparations was monitored to detect catalase activity.

### Dendritic cell culture

Murine dendritic cell line DC2.4 was cultured in Dulbecco’s Modified Eagles Medium (DMEM) with high glucose and L-Glutamine supplemented with 5% fetal calf serum, penicillin and streptomycin (100 U/ml), non-essential amino acids, and 10mM HEPES at 37°C and 5% CO_2_. Mouse bone marrow derived dendritic cells (BMDC) were cultured from the bone marrow obtained from the femurs of naïve C57BL/6J mice [28]. RPMI 1640 + GM-CSF (20ng/ml) supplemented with L-glutamine, 50 μM 2-mercaptoethanol, with antibiotics (100 U/ ml penicillin and 100 U/ml streptomycin (Pen/Strep) and 50μg/ml Gentamicin) and 10% heat inactivated FCS was used to culture BMDC [28]. The cultures were replenished every 2 days by gently swirling the plates, removing 75% of media along with non adherent granulocytes, followed by addition of fresh media with GM-CSF. Semi adherent BMDC (day 7) were harvested and used for *in vitro* DC stimulation assays as previously described [28]. All the media components for DC culture and harvest were obtained from Life Technologies, Carlsbad, CA.

### Mice immunization and challenge

At 8 weeks of age, mice (C57BL/6J) were immunized with 2 x 10^9^ CFU EBST or live attenuated AroA suspended in PBS. The immunization was performed by oral gavage with feeding needles (22 x 1^1/2^ with 1.25 mm ball; Popper & Sons, NY). Mice were diet restricted 5 h before oral infection. A SHAM control group (non-vaccinated and challenged) and naïve control group (non-vaccinated and unchallenged) were also maintained. For the EBST immunized mice, 2 booster doses consisting of 2 x 10^9^ CFU EBST were given at 2-week intervals. The mice were observed for the appearance of symptoms of infection or mortality. 14 weeks after primary immunization, mice from EBST, AroA and SHAM group were challenged via oral route with approximately 1 x 10^8^ CFU virulent nalidixic acid resistant *S*. Typhimurium strain ST 14028. At 3 days and 7 days post challenge, the mice were sacrificed and spleens were harvested for extracting splenocytes for in vitro re-stimulation assays

### *In vitro* stimulation assays

DCs (DC2.4 cells or BMDC) were seeded in 96 well U bottom tissue culture plate (BD, Franklin Lakes, NJ) at 2 x 10^5^ cells per well. DCs were co-incubated with indicated titrations of stimulants such as EBST, HKST, and live ST. A commercial ST vaccine (Salmune®, Cevabiomune, Lenexa) formulation was also included as a control for BMDC stimulation. Plates with DCs pulsed with the stimulants were incubated at 37°C, 5% CO_2_ for 24 h. For intracellular cytokine staining (ICS), the DCs were co-incubated with all the above mentioned stimulants along with 1/1000 dilution of protein transport inhibitor containing Brefeldin A (BD GolgiPlug™, BD Biosciences, San Jose, CA) at 37°C, 5% CO_2_ for 4 h. Splenocytes were harvested from individual mice [29] from the EBST, AroA, and the SHAM treatment groups. Approximately, 2 x 10^6^ splenocytes were seeded per well on 96 well U bottom tissue culture plate and co-incubated with indicated titers of stimulants such as EBST, HKST or anti-murine CD3e antibody (clone 145-2C11) at 37°C, 5% CO_2_ for 24 h.

### Surface and intracellular cytokine staining

Surface staining was used to detect the upregulation of cell surface markers on DCs (DC2.4 cells or BMDC) and splenocytes in response to the stimulants. Intracellular cytokine staining (ICS) was used to detect cytokine production in response to *in vitro* stimulation. After defined incubation periods (24 h for surface staining and 4 h for ICS) with stimulants at an MOI (Multiplicity of Infection) of 1:10, DCs were washed twice with 0.5% BSA supplemented PBS (PBSA). Surface staining was performed using monoclonal anti-mouse antibodies such as Phycoerythrin (PE) labeled murine CD11c (clone N418), Allophycocyanin (APC) labeled CD40 (clone 3/23), APC labeled CD80 (clone 16-10A1), Alexa Fluor® 700 labeled CD86 (clone GL1), eFluor® 450 labeled MHCII (clone AF6-120.1). Surface stained cells were fixed using 2% paraformaldehyde and permeabilized with Perm/wash buffer. Monoclonal anti-mouse antibodies against proinflammatory cytokine TNF, Alexa Fluor® 700 labeled TNF (clone MP6-XT22) were used for staining the permeabilized DCs. Stained DCs were further washed and re-suspended in PBSA and stored at 4°C in dark until flow cytometry analysis.

In case of *in vitro* restimulation of splenocytes, Brefeldin A was added at 1/1000 dilution during the final 6 h of incubation. After 24 h of incubation, splenocytes were washed twice with PBSA and surface stained in the presence of Fc block (clone 2.4G2) with monoclonal anti mouse antibodies Pacific Blue™ labeled CD4 (clone RM4-5) and FITC labeled CD8a (clone 53–6.7). Following staining, splenocytes were fixed and permeabilized with Cytofix/Perm buffer. Monoclonal anti mouse antibodies Alexa Fluor® 647 labeled IFNγ (clone XMG1.2) and PE labeled TNF (clone MP6-XT22) were used to stain the permeabilized splenocytes for detecting the cytokines. Stained cells were finally washed and resuspended in PBSA and stored at 4°C in the dark. For all the *in vitro* stimulation assays, unstimulated and unstained cells were maintained as controls. All flow cytometry reagents and antibodies were purchased from BD Pharmingen, San Jose, CA. Multi-parameter flow cytometry was performed using BD FACSAria™II (BD, Franklin Lakes, NJ) and data acquired with BD FACSDiva™ software (BD, Franklin Lakes, NJ). Flow cytometric data analysis was carried out with FlowJo software (Tree Star, San Carlos, CA).

### Immune modulator stability

The potential of EBST to induce maturation of DCs was used as a parameter to assess the immune modulator’s potency upon storage at different temperature conditions over a set period of time. After eBeam irradiation, EBST samples were stored at room temperature, 4°C, -20°C for up to 6 months. Lyophilized EBST was also stored at -20°C for 6 months. Two independent lots of eBeam-inactivated samples were stored. The potency of the EBST lots were tested monthly using *in vitro* DC stimulation assays with DC2.4 cell line.

### Statistical analysis

Unless otherwise stated all data graphs and statistical analysis were generated using GraphPad Prism version 8.0 (GraphPad Prism software, La Jolla, CA). A survival curve was plotted to calculate the eBeam inactivation kinetics of *S*. Typhimurium. Plate counts (log_10_ values) were plotted as a function of measured eBeam dose (kGy) and linear regression analysis was performed. The negative reciprocal of the slope was reported as the D_10_ value. Kruskal-Wallis one-way ANOVA was used for statistical comparison of bacterial colonization between different treatment mice groups. In case of *in vitro* stimulation assays, a two tailed student’s t-test was used to compare different treatment mice groups for each antigen at indicated time points. For all the statistical analysis significance was set at p ≤ 0.05.

## Results

### eBeam inactivation of *S*. Typhimurium

**Fig 1:**
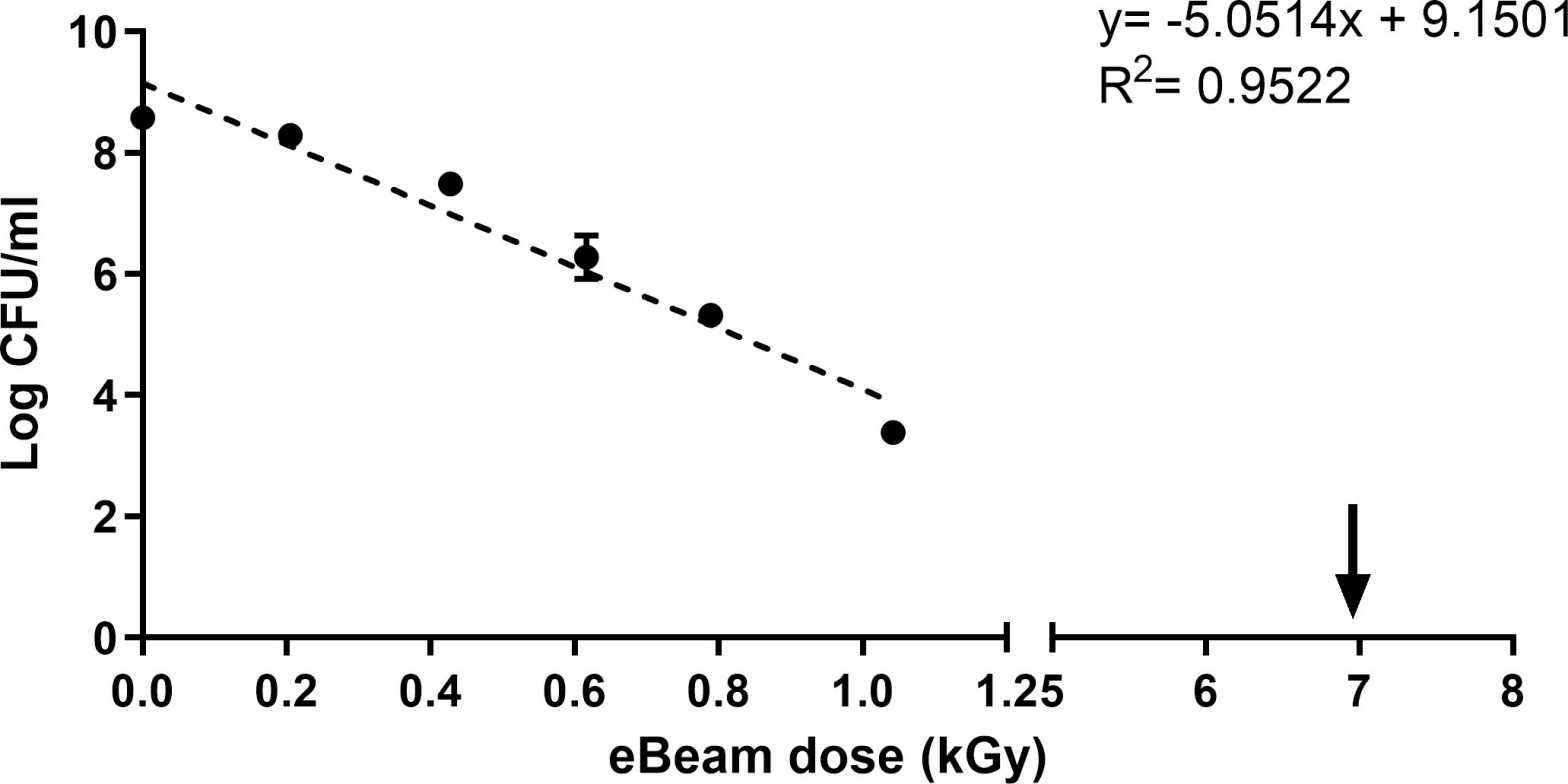
Electron beam irradiation inactivates *S*. Typhimurium. Inactivation kinetics of *S*. Typhimurium exposed to different doses of eBeam irradiation. The D_10_ value (0.19 kGy) was calculated using the negative reciprocal of regression slope of the inactivation curve. The arrow represents the lethal eBeam dose used for preparing the eBeam immune modulation formulation (EBST).

Increasing eBeam doses resulted in a log linear inactivation of *S*. Typhimurium (ST) (Fig 1). The dose required for achieving 90% reduction (D_10_ value) of ST in PBS was calculated as 0.19 kGy. Complete inactivation of bacterial cultures (~1x 10^9^ CFU/ml) was achieved at higher eBeam doses of 6 kGy, 7kGy, and 8 kGy. There was no growth of eBeam (7 kGy) inactivated ST in TSB media when incubated up to 10 days at either 37°C and room temperature. Microscopic counts of EBST did not increase, even after 2 days in TSB. Mice orally gavaged with EBST did not shed *Salmonella* sp. for up to 14 days post inoculation (Table 1A). The liver, spleen, MLN and cecum were negative for *Salmonella* sp. 14 days post inoculation (Table 1B). Absence of viable *Salmonella* colonies confirmed the inability of EBST to regrow under favorable *in vivo* conditions.

**Table 1:** Inability of eBeam-inactivated *S*. Typhimurium (EBST) to multiply *in vivo* in a *Salmonella* susceptible mice (C57BL/6J) model.

**(a) *Salmonella* recovery from fecal pellets (FP)**			
**Days of FP collection**	**Sham mice**	**Live ST mice (Log CFU/g FP)**	**EBST mice**
Day 3	BD*	4.79 ± 0.49	BD*
Day 7	BD*	4.79 ± 0.23	BD*
Day 10	BD*	4.61 ± 0.48	BD*
Day 14	BD*	4.46 ± 0.51	BD**
**(b) *Salmonella* recovery from organs**			
**Organ**	**Sham mice**	**Live ST mice (Log CFU/organ)**	**EBST mice**
Liver	BD^#^	2.24 ± 1.12	BD^#^
Spleen	BD^#^	2.07 ± 1.03	BD^#^
Cecum	BD^#^	4.73 ± 2.36	BD^#^
MLN	BD^ɸ^	3.31 ± 1.65	BD^ɸ^

*, BD = Below detection limit of 1.69 log CFU.

#, BD = Below detection limit of 1.17 log CFU.

ɸ, BD = Below detection limit of 0.69 log CFU.

### EBST maintains an intact cell membrane

**Fig 2:**
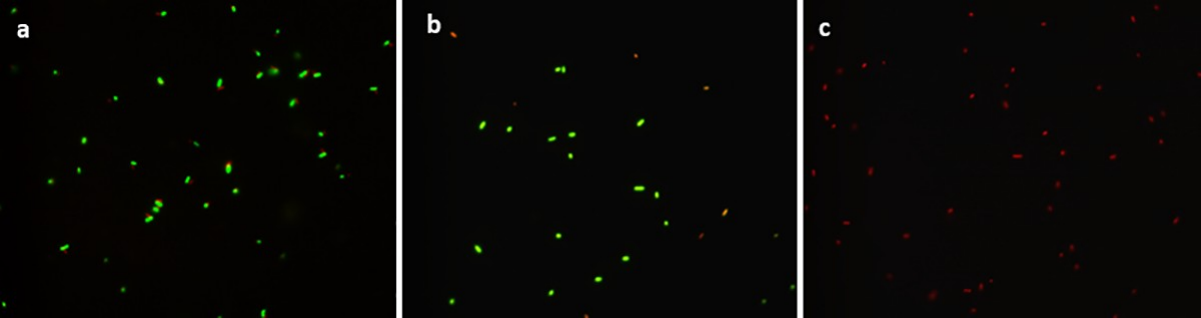
Electron beam-inactivated ST (EBST) maintains cell membrane integrity. Membrane integrity of *S*. Typhimurium remains unaffected by eBeam irradiation. Staining with Live/ Dead BacLight kit reveals the presence of intact cell membrane for (A) Live ST and (B) EBST indicated by the green labelled bacterial cells. Red colored staining of (C) HKST indicates disrupted cell membrane due to heat treatment.

**Fig 3:**
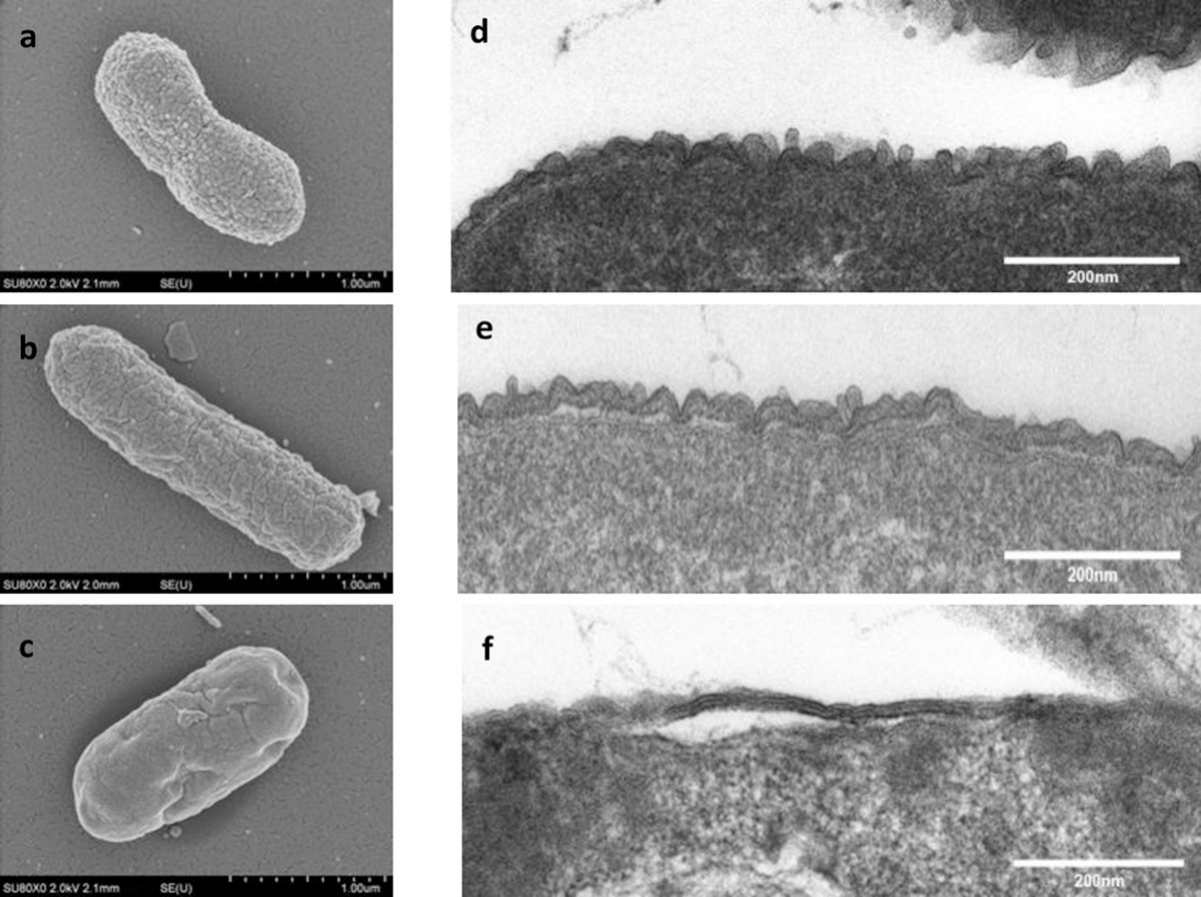
Membrane morphology of EBST is similar to live ST. Scanning electron microscopic analysis shows presence of intact bacterial cell membrane for (A) Live ST and (B) EBST. (C) HKST appears to be morphologically uneven with shrunken cell membrane. Transmission electron micrographs of (D) live ST (E) EBST and (F) HKST reveals the presence of surface molecules on bacterial cell membrane of both live and EBST. HKST shows a smoothened surface indicative of loss of surface molecules.

EBST maintained intact cell membranes similar to live ST, indicated by green fluorescent bacterial cells (Fig 2A and 2B). The damaged cell membrane from heat inactivation is depicted in Fig 2C. The EBST showed no discernable differences when examined by SEM compared to live ST controls (Fig 3A and 3B). A surface “smoothening” was observed in case of HKST (Fig 3C). TEM analysis revealed that EBST and live ST had quite similar topology (Fig 3D and 3E). The membrane damage is clearly evident in the HKST preparation (Fig 3F). The HKST membrane appears smoothed out and detached from the underlying periplasm in comparison to the live and EBST (Fig 3F). The cell content of HKST also appear to be disorganized.

### Immunoreactivity profile of EBST proteins

**Fig 4:**
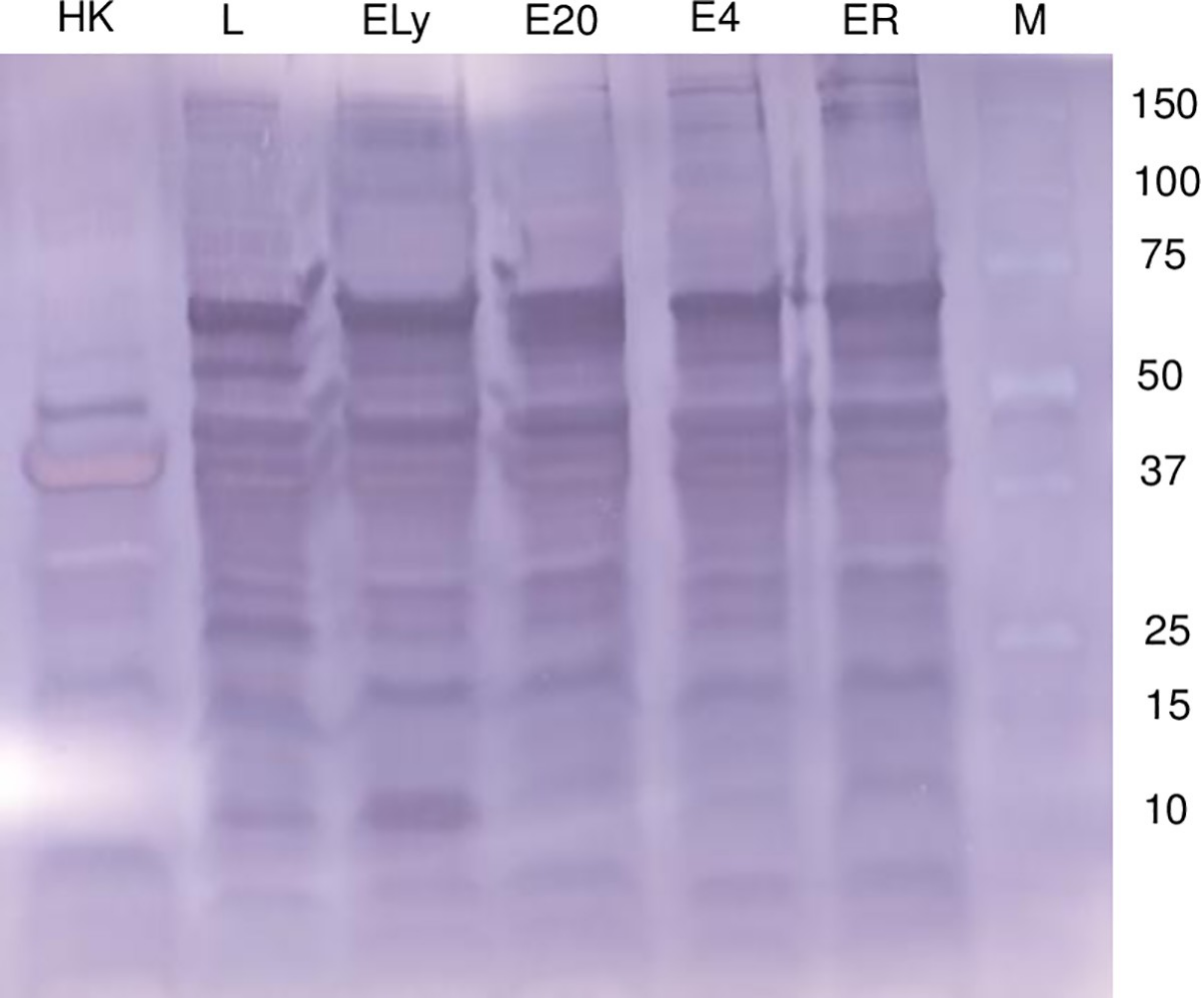
EBST retains immunogenic proteins that are detected by live ST immune mice serum. EBST stored at room temperature (ER), 4°C (E4), -20°C (E20), lyophilized (ELy), Live ST (L), heat killed ST (HK), Protein ladder (M). After eBeam-inactivated, ST was stored at multiple temperature conditions such as 4°C, -20°C, and room temperature and as lyophilized preparation. Total extracted proteins (20 μg) were separated using SDS-PAGE and analyzed by western blotting. Immunodetection was carried out using 1/1000 dilution of *S*. Typhimurium infected mice serum as the primary antibody and 1/ 20,000 dilution of alkaline phosphatase conjugated sheep anti mouse IgG F(ab`)_2_ fragment as secondary antibody.

The immunoreactivity profile of EBST was very similar to that of live ST (Fig 4). A marked difference in the banding pattern was observed in case of HKST immunoreactivity profile in comparison to live and EBST. Proteins extracted from EBST stored at different temperature conditions such as room temperature, 4°C, -20°C, and lyophilized EBST were also analyzed to detect the *Salmonella* specific protein profiles. Storage at different temperatures did not induce any alteration in the immunoreactivity profiles of EBST (Fig 4).

### EBST retains metabolic activity

**Fig 5:**
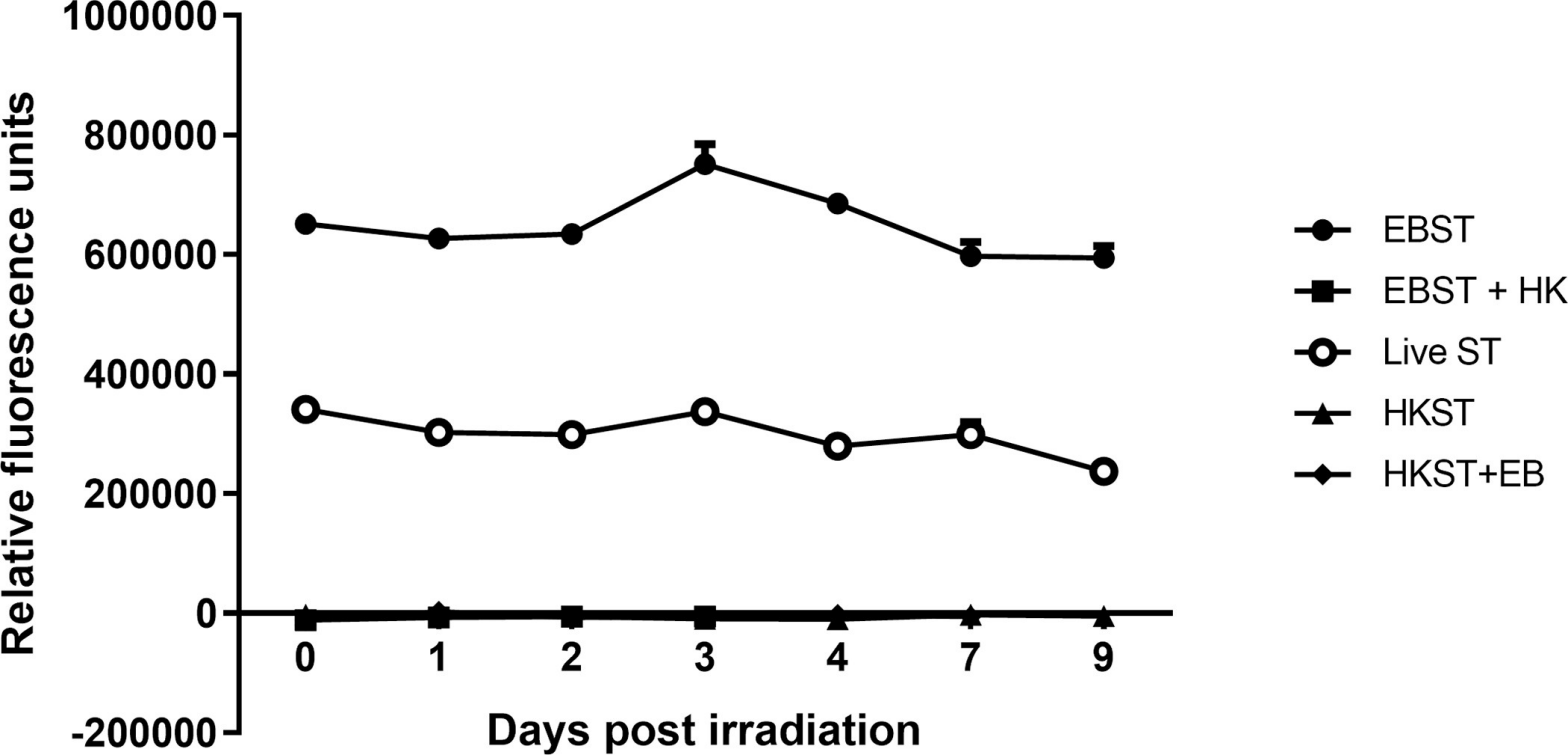
EBST remains metabolically active and retains the activity for extended time periods of storage at 4°C. Metabolic activity of *S*. Typhimurium post eBeam irradiation was measured using Alamar blue assay. The reduced environment present in metabolically active cells are detected by redox indicator which fluoresces. The fluorescence readings obtained for EBST, live ST and heat killed ST (HKST) was measured on a daily basis up to 9 days and are reported as line graph. Metabolic activity of EBST cells followed by heat inactivation (EBST+HK) and HKST followed by eBeam irradiation (HKST+EB) were measured as process controls.

Fluorescence readings obtained from the Alamar Blue® assay indicate that EBST were metabolically active even after 10 days of storage at 4°C (Fig 5). The EBST cells exhibited higher metabolic activity compared to live ST whereas, HKST showed no residual metabolic activity. Presence of metabolic activity in EBST was further confirmed with biochemical assays. Phenol Red Broth supplemented with sucrose when inoculated with EBST changed from red to yellow color (Table 2) suggesting acid production via sugar fermentation by EBST. Despite the color change, EBST inoculated media lacked turbidity and typical gas formation normally present in live ST inoculated media. Bubble formation was observed in the catalase test EBST similar to live ST (Table 2).

**Table 2:** eBeam-inactivated *S*. Typhimurium (EBST) remains metabolically active and tests positive for standard biochemical and enzymatic assays.

Assay	Parameters	Live ST	HKST	EBST
**Carbohydrate (Sucrose) utilization assay**	Media color change	+	-	+
	Gas production	+	-	-
	Turbidity	+	-	-
**Catalase assay**	Bubble formation	+	-	+

Live ST: Live *S*. Typhimurium.

HKST: Heat killed *S*. Typhimurium.

EBST: eBeam treated *S*. Typhimurium.

### EBST induces maturation of dendritic cells

**Fig 6:**
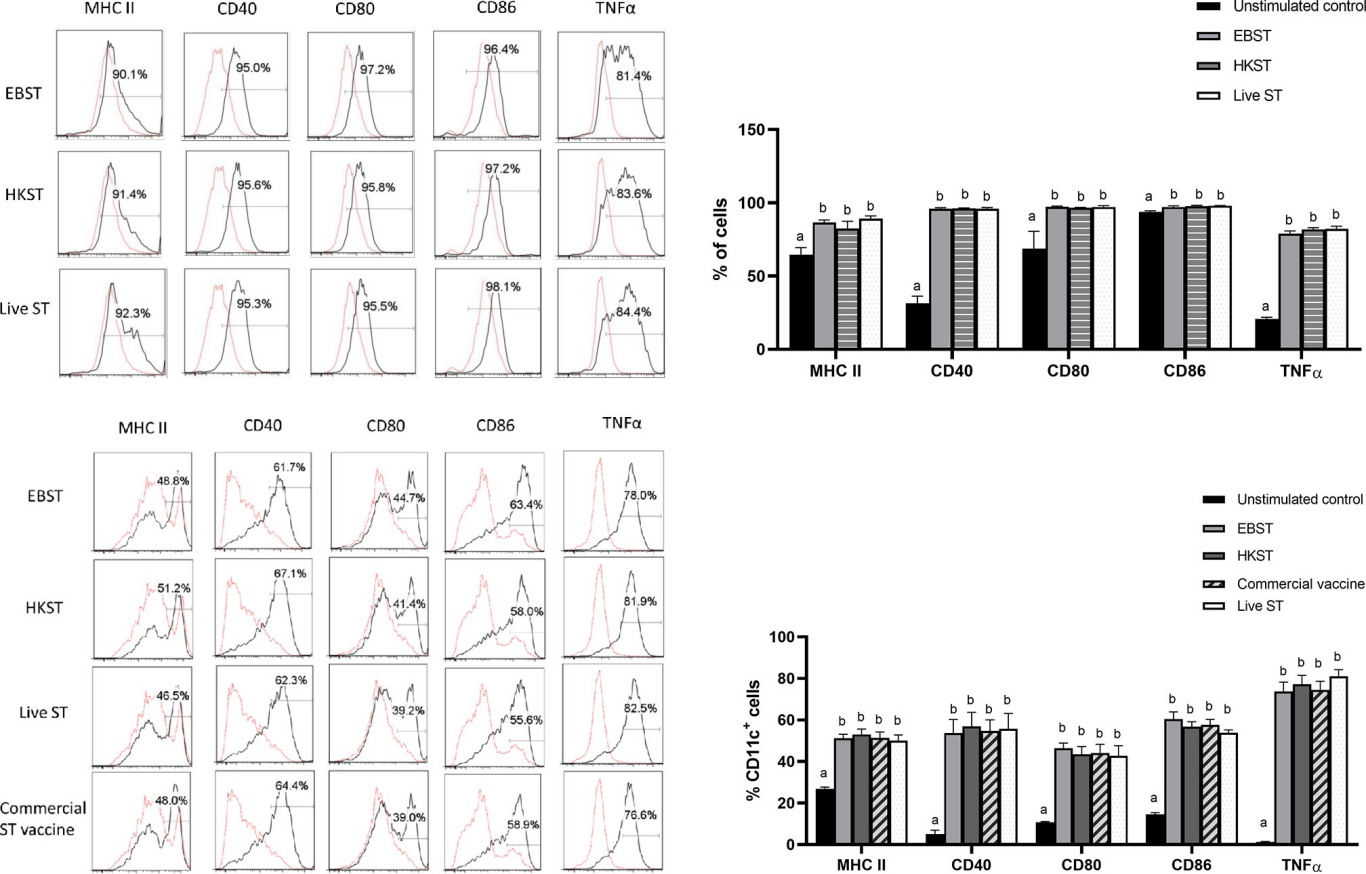
EBST induces efficient DC maturation and triggers proinflammatory cytokine production in DC2.4 cells and BMDC. DC2.4 cells were coincubated with EBST, HKST and live *S*. Typhimurium (MOI 1:10 for 24 h for surface markers and 4h for proinflammatory cytokine) as indicated to the left side of each row of histograms (Fig 6A). Each column of histogram represents expression level of surface markers MHC-II, CD40, CD80, CD86 and proinflammatory cytokine TNFα. Percentages in the gated region indicate the proportion of DC expressing high levels of various surface markers and TNFα. Expression levels of unstimulated DC (dotted red line) is compared to the antigen stimulated (thin black line) DC2.4 cells. Data are representative of three independent experiments. Bar graph summarizes the results from three independent experiments (Fig 6B). BMDCs were coincubated with EBST, HKST, live *S*. Typhimurium and a commercially available live attenuated ST vaccine (MOI 1:10 for 24 h for surface markers and 4h for proinflammatory cytokine) as indicated to the left side of each row of histograms (Fig 6C). Each column of histogram represents expression level of surface markers MHC-II, CD40, CD80, CD86 and proinflammatory cytokine TNFα. Percentages in the gated region indicate the proportion of CD11c^+^ DC expressing high levels of various surface markers and TNFα. Expression levels of unstimulated DC (dotted red line) is compared to the antigen stimulated (thin black line) DC. Data are representative of three independent experiments. Bar graph summarizes the results from three independent experiments (Fig 6D). Bars with differing letters indicate significant (p<0.05) differences among groups for each individual activation marker.

Ability of EBST to trigger DC maturation was tested *in vitro* using two murine DC models- DC2.4 cell line and primary DC culture derived from bone marrow of C57BL/6J mice (BMDC). The DC2.4 cells were stimulated with EBST, HKST and live S. Typhimurium for 24 h and the surface expression of MHC-II, CD40, CD80 (B7-1) and CD86 (B7-2) was analyzed. Production of proinflammatory cytokine TNFα was measured followed by 4 h of co-incubation with stimulants. EBST induced efficient DC maturation indicated by the up-regulation of MHC-II, CD40, CD80 and CD86 surface expression on DC2.4 (Fig 6A). The level of DC maturation induced by EBST was similar to live ST and HKST. Increased production of TNFα by DC2.4 cells is indicative of the potent proinflammatory properties of EBST. For BMDC activation studies, a commercial live attenuated ST vaccine formulation (Salmune®, Ceva biomune, Lenexa, KS) was also included as a stimulant for a comparative analysis with EBST. Results from BMDC activation studies supported the findings from DC2.4 cell line studies. When compared to the unstimulated media control, co-incubation of BMDC with stimulants generated a clear shift towards DC population expressing higher levels of MHC-II, CD40, CD80, CD86 and TNFα (Fig 6C). These results demonstrate that EBST induced maturation and activation of DC were similar to that induced by commercial live attenuated vaccine formulation.

### EBST induces antigen specific cellular immune responses

**Fig 7:**
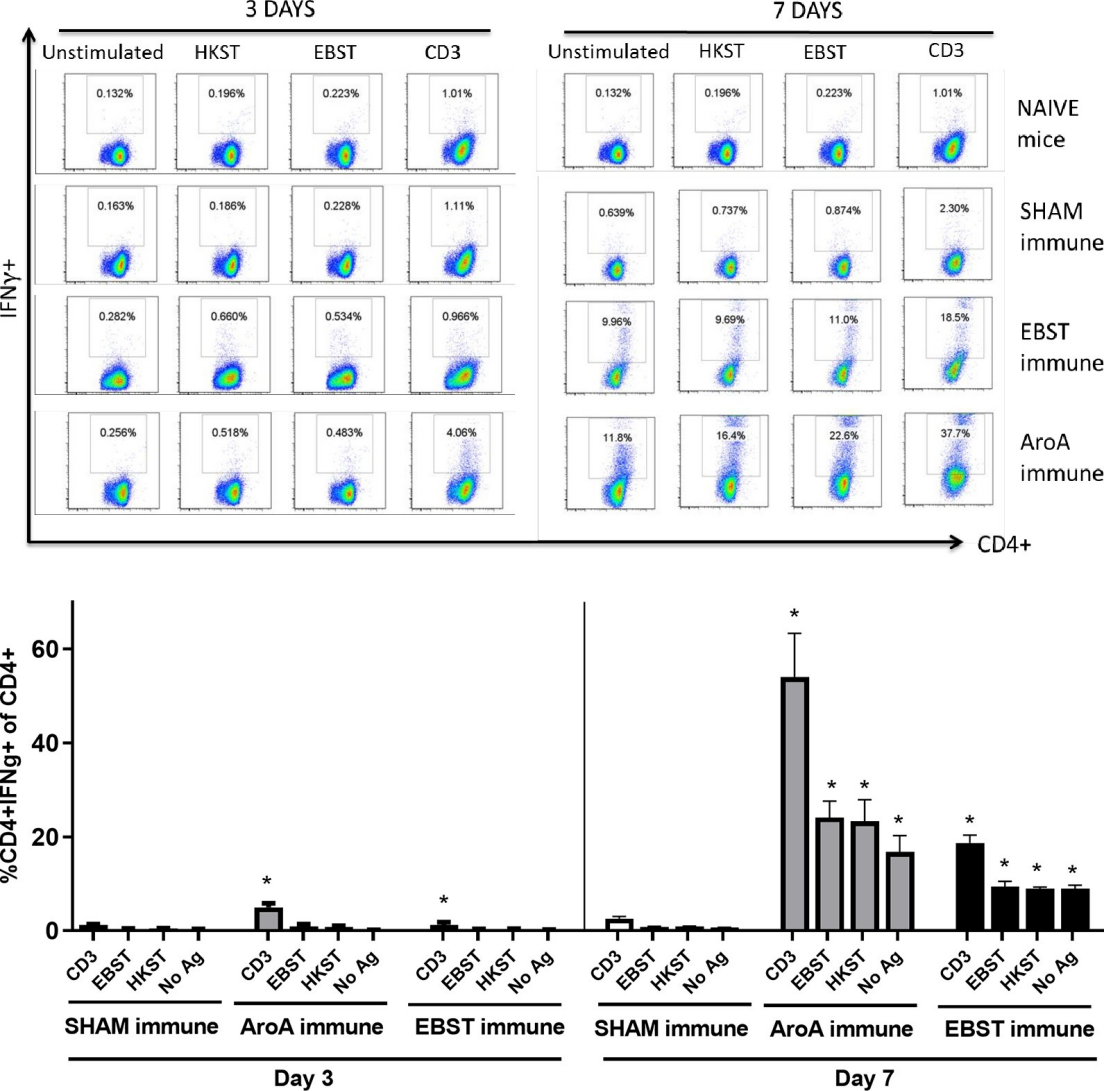
EBST immune mice exhibit *Salmonella* specific CD4^+^IFNγ^+^ T cell responses during virulent ST challenge. Individual EBST immune, AroA immune, and Sham immune mice were infected with virulent ST and at 3 days and 7 days post infection. Splenocytes were measured for production of IFNγ by antigen specific CD4^+^ T cells using intracellular cytokine staining (ICS). Antigens used were EBST and HKST (2 x10^7^ cells/ well) and anti-CD3 antibody as a positive control and unstimulated splenocytes as a negative control. Percentages in the gated region of dot plots indicate the proportion of CD4^+^IFNγ^+^ of total CD4^+^ splenocytes (Fig 7A). Data are representative of three-five individual mice per group. The bar graph data represents mean ± SEM of 3–5 mice per group. The Y axis represents percentage of CD4^+^IFNγ^+^ of total CD4+ splenocytes (Fig 7B) on days 3 and 7. Statistics were determined for comparisons between group for each antigen at the indicated day. *, p≤0.05.

**Fig 8:**
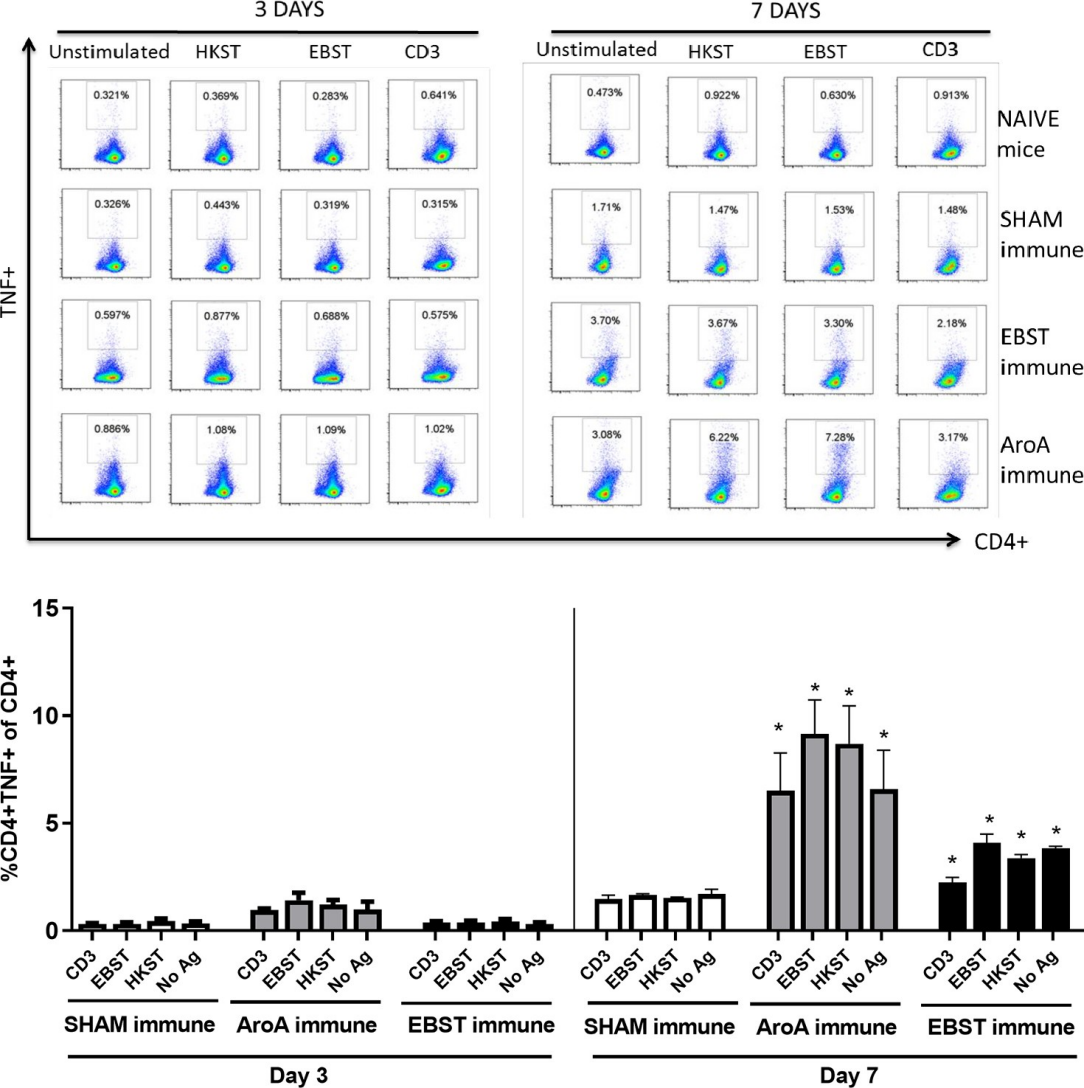
EBST immune mice exhibit *Salmonella* specific CD4^+^TNF^+^ T cell responses during virulent ST challenge. Individual EBST immune, AroA immune, and Sham immune mice were infected with virulent ST and at 3 days and 7 days post infection splenocytes were measured for production of TNFa by antigen specific CD4^+^ T cells using intracellular cytokine staining (ICS). Antigens used were EBST and HKST (2 x10^7^ cells/ well) and anti-CD3 antibody as positive control. Unstimulated splenocytes were included as control. Percentages in the gated region of dot plots indicate the proportion of CD4+TNF+ of total CD4+ splenocytes (Fig 8A) The data represents three-five individual mice per group. The bar graph data represents the mean ± SEM of 3–5 mice per group. Y axis represents percentage of CD4^+^TNF^+^ of total CD4^+^ splenocytes (Fig 8B) on days 3 and 7. Statistics were determined for comparisons between group for each antigen at the indicated day. *, p≤0.05.

**Fig 9:**
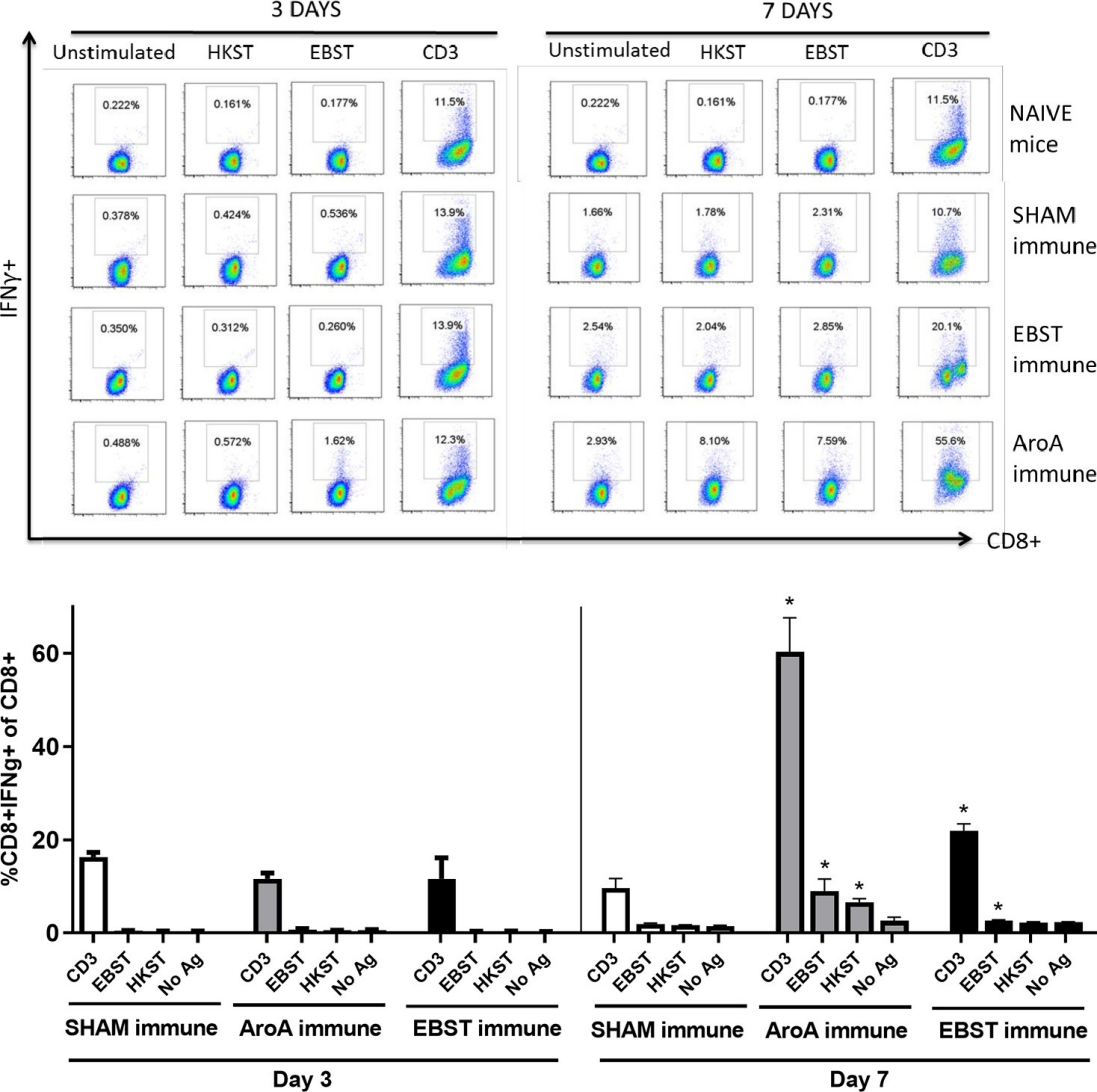
*Salmonella* specific CD8+IFNγ+ T cell responses in EBST, Live ST and Sham mice during virulent ST challenge. Individual EBST immune, AroA immune, and Sham immune mice were infected with virulent ST and at 3 days and 7 days post infection splenocytes were measured for production of IFNγ by antigen specific CD8+ T cells using intracellular cytokine staining (ICS).Antigens used were EBST and HKST (2 x10^7^ cells/ well) and anti-CD3 antibody as positive control. Unstimulated splenocytes were included as control. Percentages in the gated region of dot plots indicate the proportion of CD8+IFNγ+ of total CD8+ splenocytes (Fig 9A). The data represents three-five individual mice per group. The bar graphs represents mean ± SEM of 3–5 mice per group. Y axis represents percentage of CD8+IFNγ+ of total CD8+ splenocytes (Fig 9B) on days 3 and 7. Statistics were determined for comparisons between group for each antigen at the indicated day. *, p≤0.05.

**Fig 10:**
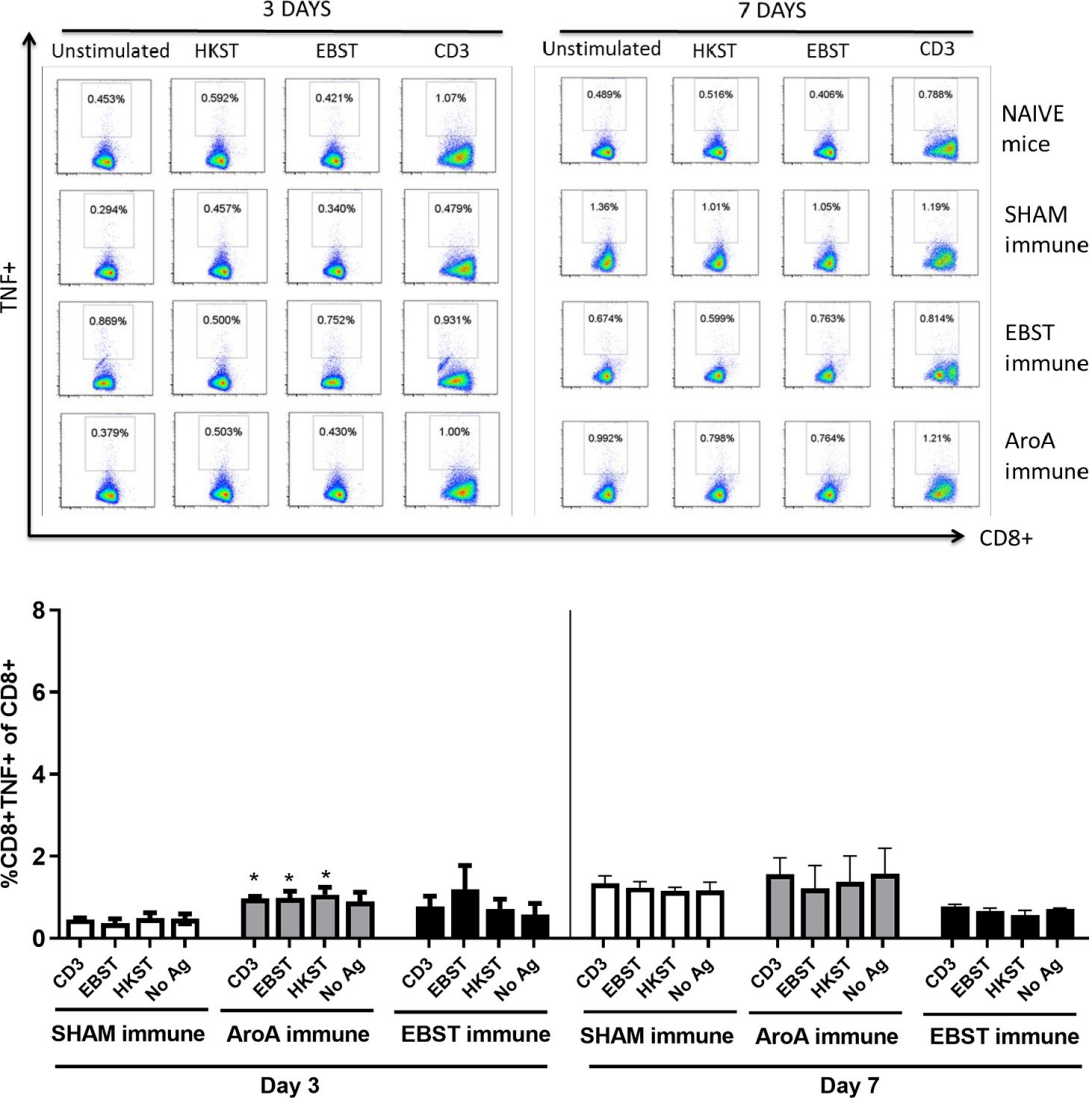
*Salmonella* specific CD8+TNF+ T cell responses in EBST, Live ST and Sham mice during virulent ST challenge. Individual EBST immune, AroA immune, and Sham immune mice were infected with virulent ST and at 3 days and 7 days post infection splenocytes were measured for production of TNFa by antigen specific CD8+ T cells using intracellular cytokine staining (ICS). Antigens used were EBST and HKST (2 x10^7^ cells/ well) and anti-CD3 antibody as positive control. Unstimulated splenocytes were included as control. Percentages in the gated region of dot plots indicate the proportion of CD8+TNF+ of total CD8+ splenocytes (Fig 10A). The data represents three-five individual mice per group. The bar graph represents mean ± SEM of 3–5 mice per group. Y axis represents percentage of CD8+TNF+ of total CD8+ splenocytes (Fig 10B) on days 3 and 7. Statistics were determined for comparisons between group for each antigen at the indicated day. *, p≤0.05.

Live attenuated ST vaccines are supposedly more effective than inactivated vaccines in mice models, due to their ability to induce strong cell-mediated immunity [2, 20–22]. Therefore, the ability of EBST immunized mice to develop antigen specific cellular immune response during virulent *Salmonella* challenge was tested. Mice were orally immunized with EBST, AroA ST and PBS (Sham immune). EBST immune mice were boosted twice at 2 weeks intervals via same route. All groups of mice were challenged with virulent ST, 14 weeks after primary immunization and spleens were harvested at days 3 and 7 post challenge. Frequencies of CD4^+^ and CD8^+^ T cells that produced cytokines IFNγ and TNF were measured after *ex vivo* re-stimulation of splenocytes with total Salmonella antigens (EBST and HKST) for 24 h. Measuring the frequency of IFNγ producing T cells provides a direct indication of the Th1 mediated immune response. In naïve and sham immune mice, frequencies of CD4^+^ T cells producing IFNγ^+^ and TNF^+^ spontaneously (CD3) or in response to antigen stimulation were found to be low on day 3 and day 7 post challenge. However, on day 7 post challenge, both EBST and AroA immune mice showed increased frequencies of CD4^+^IFNγ^+^ and CD4^+^TNF^+^ T cells that responded to Salmonella antigens (Figs 7 and 8). Compared to sham immune mice, EBST immune mice splenocytes had significantly high frequency of *Salmonella* specific CD4^+^IFNγ^+^ (p = 0.016) and CD4^+^TNF^+^ (P = 0.0043) T cells. Both AroA and EBST immune mice showed an increase in antigen specific CD4^+^IFNγ^+^ and CD4^+^TNF^+^ T cells from day 3 to day 7 post challenge. Frequency of antigen specific CD8^+^ T cells producing IFNγ was far lower compared to CD4^+^ T cells (Fig 9). Antigen specific CD8^+^ IFNγ^+^T cells were higher in AroA immune mice splenocytes compared to EBST and sham immune mice (Fig 9). CD8^+^TNF^+^ T cells were found to be present in similar proportion with no significant difference between sham immune, AroA immune and EBST immune mice (Fig 10).

**Fig 11:**
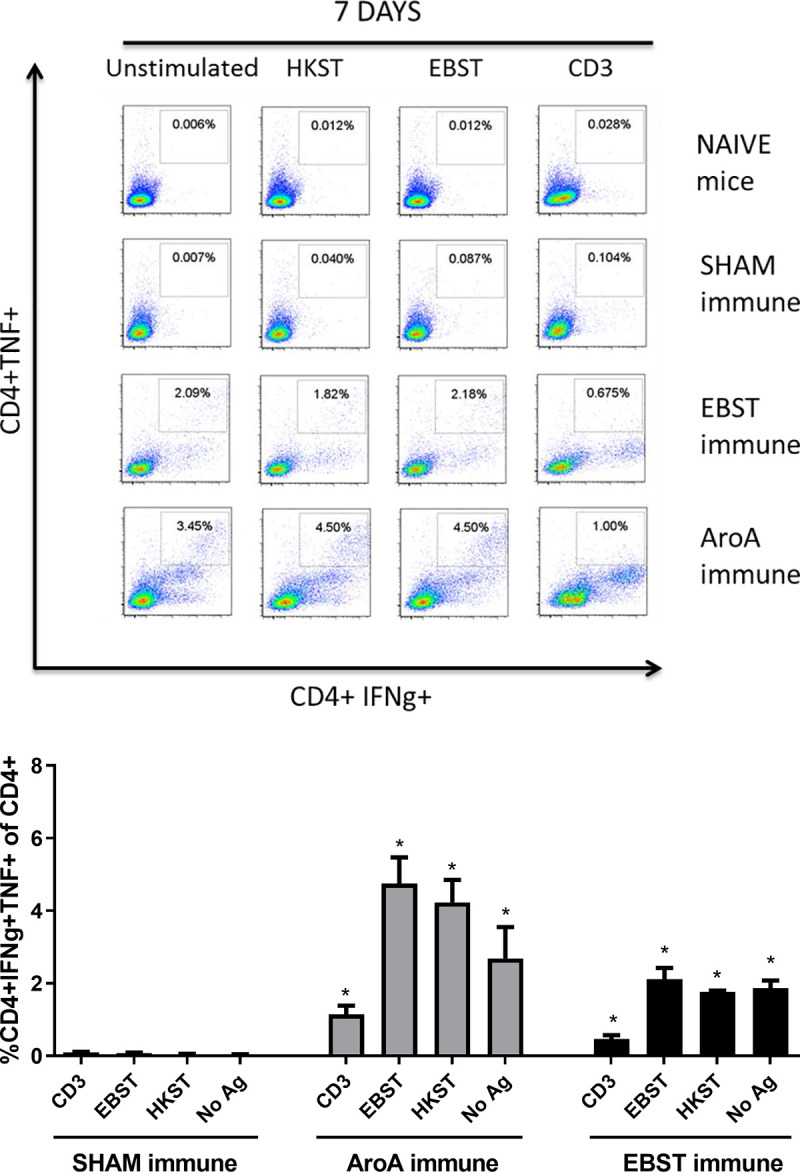
EBST immune mice exhibit *Salmonella* specific multifunctional CD4+ T cells during virulent *Salmonella* challenge. Simultaneous production of cytokine IFNγ and TNF by antigen specific CD4+ T cells was measured using multiparameter flow cytometry and intracellular cytokine staining (ICS) of splenocytes for individual EBST immune, AroA immune and Sham immune mice at 7 days post infection with virulent ST (Fig 11A). For comparison, naïve mice splenocytes were included. Antigens used were EBST and HKST (2 x10^7^ cells/well) and anti-CD3 antibody as positive control. Unstimulated splenocytes were included as control. The percentages in the gated region of dot plots indicate the proportion of CD4^+^IFNγ^+^TNF^+^ of total CD4+ splenocytes. Data are representative of three individual mice per group. The bar graphs represent mean ± SEM of 3 mice per group. Y axis represents percentage of CD4^+^IFNγ^+^TNF^+^ of total CD4+ splenocytes (Fig 11B).

Presence of multifunctional Th1 cells which can simultaneously secrete multiple cytokines such as IFNγ, TNF and IL-2 are found to be better correlates of vaccine induced protection compared to single cytokine secreting T cells (Darrah et al., 2007). The presence of CD4^+^IFNγ^+^TNF^+^ T cells in splenocytes from EBST, AroA and sham immune mice after *in vitro* restimulation with *Salmonella* specific antigens was tested using multi-parameter flow cytometry (Fig 11). It was observed that both EBST and AroA immune mice splenocytes had higher frequencies of CD4^+^IFNγ^+^TNF^+^ T cells that responded to total *Salmonella* antigens. Sham immune mice on the other hand, did not trigger stimulation of multifunctional T cells (Fig 11). EBST immune mice had significantly higher (p = 0.003) frequency of multifunctional CD4^+^ T cells compared to sham immune mice. Multifunctional CD4^+^ T cells were less stimulated in response to anti-CD3 antibody control (Fig 11).

### Immunogenic and pro-inflammatory properties during long-term storage

**Fig 12:**
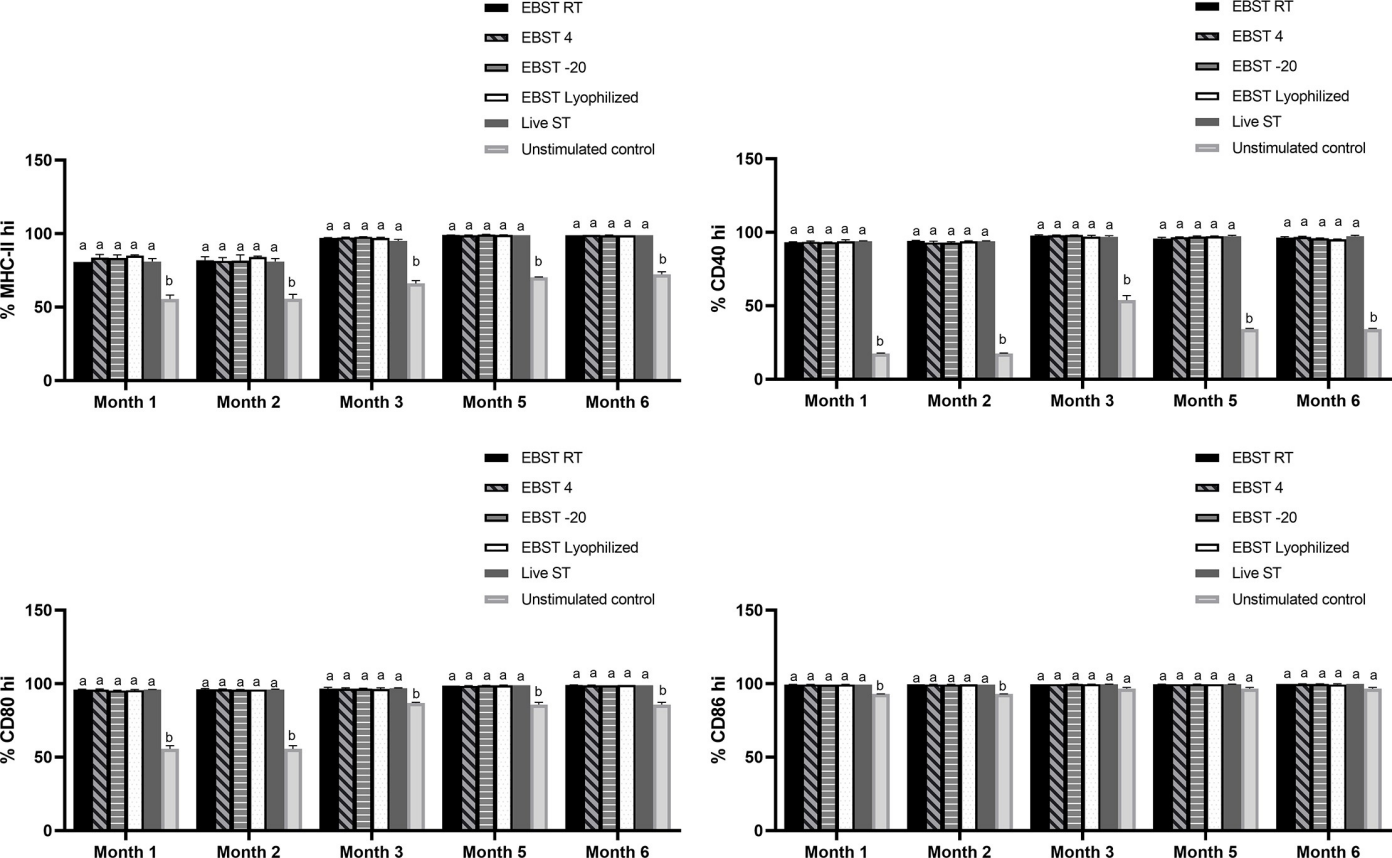
EBST remains immunogenically stable during storage at room temperature for extended time period. EBST formulation stored at different temperature conditions such as room temperature (RT), 4C, -20°C and after lyophilization induced stable maturation of DC2.4 cells indicated by the upregulation of (A) MHC-II, (B) CD40, (C) CD80, and (D) CD86 for up to 6 months of storage. Freshly prepared live *S*. Typhimurium was included as a positive control to stimulate DC2.4 cells, unstimulated DC2.4 cell provided base level expression of different surface markers on DC2.4 cells. Data represents mean values of proportion of cells positive for indicated surface marker. Bars with differing letters indicate significant (p≤0.05) differences among groups for each individual activation marker.

The ability of EBST formulation to retain the immunogenic properties during storage at different temperature conditions was tested as a measure to assess its immune modulatory potency (Fig 12A–12D). The EBST formulation was immunogenically stable as evidenced by their consistent DC maturation capability up to 6 months of storage at various temperatures (Fig 12A–12D). Expression levels of DC maturation indicators such as CD40, MHC-II, CD80 and CD86 were up-regulated and were on par with the level of live ST stimulation (Fig 12A–12D).

## Discussion

The eBeam *S*. Typhimurium (EBST) immune modulator was developed by inactivating live *S*. Typhimurium (ST) using high-energy electron beam. The eBeam dose for EBST immune modulator was based on ability to ensure complete inactivation (absence of regrowth *in vitro* or *in vivo*) and ability to preserve the immunological epitopes. The eBeam dose required for complete bacterial inactivation varies with the target bacterial concentration [25, 30]. Based on the results from the bacterial inactivation studies and regrowth assays (*in vitro* and *in vivo*), EBST formulation was generated by irradiating ~ 1x10^9^ CFU/ml of ST at 7 kGy (SD ±0.38). The criteria that the inactivated cells could not be resuscitated either in culture media or in an immunologically susceptible mouse model, render EBST as non-pathogenic per Koch’s postulate [31]. Membrane integrity analysis using BacLight assay and electron microscopy revealed that EBST maintained an intact cell membrane similar to non eBeam-inactivated live ST (Figs 2 and 3). Moustafa et al. (2011) and Hiramoto et al. (2002) have reported the presence of intact cell membrane in gamma irradiated *Brucella* sp. and *Toxoplasma gondii* respectively. This was not unexpected as bacterial inactivation by ionizing radiation is hypothesized to occur primarily through double strand breaks in DNA [32, 33]. Damage to proteins or lipids is thought to contribute only negligibly to bacterial inactivation [9, 11, 34]. Heat inactivation damages the cell membrane and causes other structural changes (Figs 2 and 3). These findings were confirmed by the immunoreactivity profile of *Salmonella* specific antigens of EBST, HKST and live ST preparations. Results showed that HKST lacked several antigenic proteins, whereas EBST had a very similar immunoreactivity profile as live ST (Fig 4). Thus, it is inferred that unlike HKST, EBST maintains cell membrane integrity and preserves the antigenic epitopes.

Irradiation of a cell not only leads to loss of reproductive integrity, but also cause disturbance in many metabolic events and cell progression. These alterations in the key metabolic processes are important to understand the chain of events leading ultimately to cell death and the involvement of cell repair and recovery processes [35]. Although eBeam irradiation prevented bacterial multiplication, EBST remained metabolically active (Fig 5). Increased fluorescence (alamarBlue™ assay), catalase activity and acid production in fermentation medium further supports metabolic activity of eBeam inactivated *S*. Typhimurium cells (Table 2). Cells surviving radiation upregulate their metabolic state to compensate for the damage incurred during exposure [35]. Studies have shown that a dose responsive and persistent increase in oxidative and nitrosative stress was found in irradiated cells as a result of higher metabolic activity in the fraction of surviving cells [35, 36]. The radiation induced oxidative stress can impact a wide range of cellular endpoints and can last for several days to weeks after initial exposure. The alamarBlue™ assay results indicate that EBST retained metabolic activity for more than 10 days, with almost 2-fold increased activity over live ST (Fig 5). These findings support previous reports of metabolic activity in gamma irradiated *Brucella* sp. [37–39] and *Toxoplasma gondii* [40]. As per the studies by Hiramoto et al. (2002) and Magnani et al. (2009), irradiation does not cause significant interference to cellular functions. Gamma irradiated cells maintained cellular oxidative function and preserved the ability for protein and nucleic acid synthesis [37, 40]. Studies have shown that, despite impairing microbial replication by DNA fragmentation, ionizing radiation leaves considerable portions of genome amplifiable [37, 41]. Thus, these inactivated cells remain metabolically active but replication incompetent [41]. Recent studies from our laboratory provided supportive evidence for significant metabolomic changes associated with critical amino acid synthesis pathways in eBeam inactivated *E*. *coli* and *S*. Typhimurium [42]. Together, these results suggest that eBeam irradiation renders *S*. Typhimurium into a metabolically active yet non culturable (MAyNC) state [37–40, 42].

The ability of DC to interact with naïve T cells following antigen uptake, determines the specificity and polarization of the T cell mediated immunity [43, 44]. Hence, the DC maturation potential of EBST was studied as the first step of immunological characterization. When encountered with appropriate inflammatory stimuli, immature DC undergoes process of maturation to develop as a fully competent APC [44, 45]. A mature DC is characterized by increased and stable surface expression of MHC molecules, upregulation of co-stimulatory molecule surface expression, as well as enhanced cytokine secretion. The results from *in vitro* DC stimulation assays using DC2.4 cell line and BMDC showed that EBST provided optimal DC maturation and activation (Fig 6), indicated by upregulation of MHC-II, CD40, CD80, CD86 and TNFα production. Interestingly, the level of expression of surface markers and cytokine secretion induced by EBST were on par with that induced by viable live ST. These results are consistent with previous studies conducted using gamma irradiated *Brucella* sp. [39] and *Listeria monocytogenes* [13], which showed similar DC activation by irradiated, heat killed and live bacterial preparations. Conflicting reports have been published in the past with respect to the requirement of bacterial viability and longer incubation time for DC maturation by *Salmonella* [44, 46–48]. The studies by Kalupahana et al (2005) emphasized upon the requirement of prolonged contact of DC with inactivated/ HK Salmonella to induce DC activation similar to live Salmonella. These findings are in agreement with the present study as higher expression levels of MHC-II and co-stimulatory markers were observed after 24 h co-incubation with EBST and HKST (Fig 6C). A commercial live attenuated ST vaccine was used as a control for BMDC maturation studies. For an appropriate comparison, DCs were stimulated with same MOI of EBST, HKST, live ST and commercial vaccine. The results suggest that EBST immune modulator without any additional adjuvant, induced DC maturation similar to the commercial ST vaccine formulation (Fig 6C). Even though HKST triggered comparable DC maturation as EBST, heat inactivation resulted in disrupted cell membrane, altered surface morphology and loss of immunogenic epitopes in HKST. Together, these results suggest that EBST serves as a superior inflammatory stimulus to effect antigen specific immune response.

Several studies have demonstrated higher level of protective immunity in mice by live attenuated *Salmonella* vaccines, compared to killed/ inactivated vaccines [19, 21–24, 44, 49]. Reports have shown that oral immunization with live attenuated AroA^-^ ST vaccine generated Th1 type cell mediated immune response, hallmarked by the increased production of Th1 cytokine IFNγ [19, 21, 24]. Whereas, immunization with inactivated vaccines such as heat killed or purified bacterial components, generated an IL-4 dominated Th2- type response [18, 23]. Our data suggest that EBST enables host to rapidly produce Th1 derived cytokines—IFNγ and TNFα, in response to the virulent bacterial infection. The activation of antigen specific IFNγ and TNFα producing CD4^+^ T cells in both EBST immune and AroA immune mice splenocytes is a clear indication of strong Th1 type cell mediated immune response. For simultaneous detection of IFNγ^+^ and TNF^+^ T cells by ICS, BrefeldinA (Golgi plug™) was added after 18 h of co-incubation, instead of 4 h (optimal for TNF production). This delay may not be optimal for detection of TNF during ICS, hence lower frequency of CD4^+^TNF^+^ T cells compared to CD4^+^IFNγ^+^ T cells. In contrast to sham immune mice, EBST and AroA immune mice had increased IFNγ and TNFα producing CD4^+^ T cells from splenocytes without added antigen stimulation. Spleens from EBST immune, AroA immune and Sham immune were colonized with the challenge strain of Salmonella. Hence, the unstimulated splenocytes from these mice harbored Salmonella, which could have provided an antigenic stimulus for secretion of IFNγ by *Salmonella* primed CD4^+^ T cells. Although, sham immune, infected mice were also colonized with *Salmonella*, very low frequencies of CD4^+^IFNγ^+^ and CD4^+^TNF^+^ T cells were found, which indicates increased antigen specific immune responses triggered due to EBST and AroA immunization [2, 50, 51].

Although we observed higher polyclonal stimulation of CD8^+^IFNγ^+^ T cells in both EBST and AroA immune mice, significant levels of antigen specific IFNγ producing CD8^+^ T cells were present only in case of AroA immune mice. In contrast to CD4^+^ T cells, CD8^+^ T cells are mostly restricted to MHC class-I molecules that predominately present endogenous cytosolic antigens processed by proteasome in APCs [51, 52] thus Salmonella antigens acquired by phagocytosis or endocytosis may not be presented on APC MHC class-I effectively. Consistent with the results of Yrlid et al. (2001), we observed that in AroA immunized mice; contribution of IFNγ production by *Salmonella* specific CD8^+^ T cells appeared to be minor compared to CD4^+^ T cells (Fig 10). Based on these findings, we can infer that EBST is capable of inducing Th1 type cellular immune response similar to live attenuated *Salmonella* vaccines. Therefore, unlike typical killed or inactivated vaccines [21, 23] mice immunized with EBST develops robust *Salmonella* specific T cell responses.

Though the production of IFNγ or effector cytokine TNF by CD4^+^ T cells is considered necessary to mediate protection, using it as a single immune parameter to predict protection may not be sufficient [53]. As IFNγ and TNF act synergistically to mediate pathogen control, use of multifunctional CD4^+^ T cells capable of simultaneously secreting IFNγ and TNF is considered a better correlate of protection [53]. Therefore, multi-parameter flow cytometry was used to assess the frequency of multifunctional CD4^+^ T cells simultaneously producing IFNγ and TNF. Increased *Salmonella* specific multifunctional CD4^+^IFNγ^+^TNF^+^ T cells were observed in EBST immune and AroA immunized mice (Fig 11). Notably, nonspecific polyclonal stimulation of multifunctional CD4^+^ T cells were not observed in both EBST and AroA immune mice, which may signify the specificity of CD4^+^IFNγ^+^TNF^+^ T cells towards *Salmonella* antigens. EBST immune mice had significantly higher frequency of multifunctional CD4^+^ T cells compared to sham immune mice, but were relatively lower than AroA immune mice (Fig 11). In future, more defined challenge studies need to be conducted with multiple immune modulating dose regimes and challenge titers to definitively evaluate the protective immunity induced by EBST immune mice in comparison to mice immunized with live attenuated Salmonella vaccine.

Appropriate storage of vaccines plays a key role in the success of immunization. Vaccines exposed to temperatures outside the recommended range can have reduced potency and protection [1]. Hence, it is important to maintain a temperature-controlled vaccine supply chain which ultimately increases vaccine costs. The eBeam based immune modulators may provide for a rather inexpensive alternative to current vaccine formulations, especially with respect to storage and transportation without the requirement of cold chain. The potency of EBST over extended storage period was determined using *in vitro* DC2.4 activation model system. EBST retained stable immunogenic properties such as increased surface expression of DC maturation markers and production of proinflammatory cytokine, for several months at room temperature, 4°C, -20°C and also after lyophilization (Fig 12). These properties are considered to be of high value in any vaccine formulation [54]. The EBST provided consistent and stable upregulation of DC maturation markers similar to live ST. The EBST’s stability at room temperature or as lyophilized powder for extended storage period demonstrates immense cost saving potential of such formulations.

Use of eBeam irradiation for vaccine production is a relatively novel field of research. Much work has been done by various research groups across the globe with regard to the development of gamma irradiated vaccines [13–15, 38, 39, 55]. Although the basic mechanism by which eBeam and gamma irradiation inactivate microorganisms is similar [9], there are fundamental differences between eBeam and gamma irradiation with respect to irradiation source, energy and dose-rate [9, 25] as well as how the microbial cells respond. During eBeam irradiation, microbial pathogens experience ionizing radiation at significantly higher dose rate (usually in seconds) conditions compared to gamma irradiation (minutes or hours), hence eBeam has a very short processing time compared to gamma. The technology is also ideal for in-line integration into current vaccine production lines. Very importantly, eBeam technology is an ideal alternative to radioisotope-based technology which has nuclear security issues.

## Conclusion

Overall, eBeam inactivation preserved the potent proinflammatory and immunogenic properties of *S*. Typhimurium. EBST remained metabolically active yet unable to multiply *in vitro* and *in vivo*. Increasing numbers of immunocompromised population who suffer from congenital immunodeficiency, immunosuppressive therapies, HIV patients are at risk of vaccine induced diseases after immunization with replicating vaccines [4, 5]. Being a non-replicating immune-modulator provides added advantage for immunizing immunocompromised individuals. Historically, non-replicating inactivated vaccines fail to induce adequate immune response, especially against pathogen requiring cell mediated immune response [23, 53, 54, 56]. But, metabolically active EBST induce substantial *Salmonella* specific T cell responses, thus playing a meaningful role in host response during infection. The eBeam based immune modulation serves as a promising route for commercial vaccine development as the immunogenic properties of EBST remain stable during storage at room temperature or after lyophilzation for extended time period.

## Supporting information

S1 Fig (TIF)
