# Supplementary figures and images for: Assessment of microbiological correlates and immunostimulatory potential of electron beam inactivated metabolically active yet non culturable (MAyNC) Salmonella Typhimurium
Source: PLoS One. 2021 Apr 16;16(4):e0243417. doi: 10.1371/journal.pone.0243417 (PMC8051754; doi:10.1371/journal.pone.0243417)

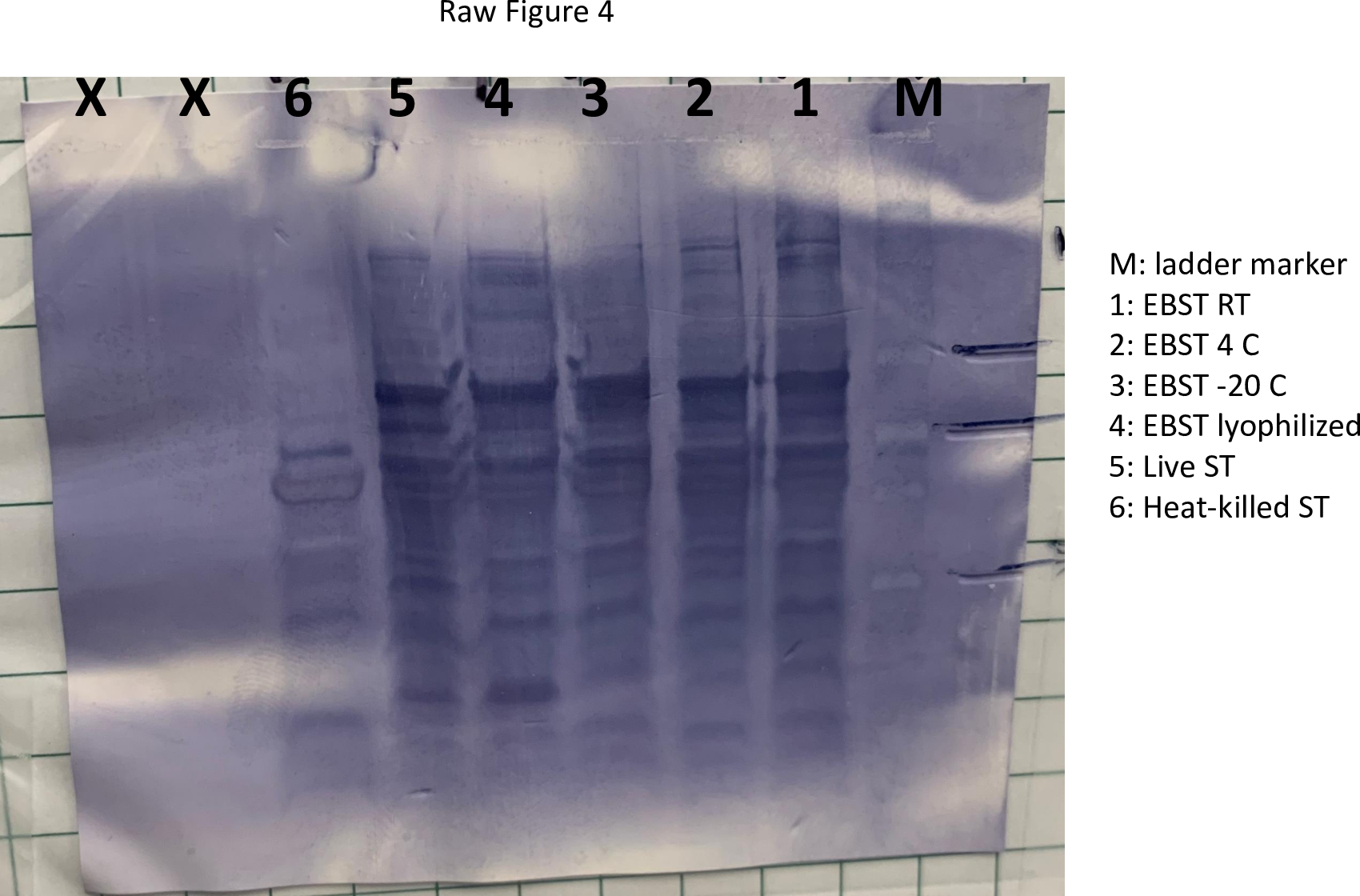

Supplement: S1 Fig — (TIF) [file pone.0243417.s001.tif]
